# Rehabilitation of Memory following Brain Injury (ReMemBrIn): study protocol for a randomised controlled trial

**DOI:** 10.1186/1745-6215-16-6

**Published:** 2015-01-06

**Authors:** Roshan das Nair, Nadina B Lincoln, Deborah Ftizsimmons, Nicola Brain, Alan Montgomery, Lucy Bradshaw, Avril Drummond, Catherine Sackley, Gavin Newby, Jim Thornton, Sandip Stapleton, Anthony Pink

**Affiliations:** Division of Rehabilitation and Ageing, Queens Medical Centre, University of Nottingham, Nottingham, NG7 2UH UK; Dept of Clinical Psychology & Neuropsychology, Nottingham University Hospitals NHS Trust, Nottingham, NG7 2UH UK; Swansea Centre for Health Economics, College of Human and Health Sciences, Swansea University, Singleton Campus, Swansea, SA2 8PP UK; Rehabilitation Medicine, Level 1, Royal Derby Hospital, Derby Hospitals NHS Foundations Trust, Derby, DE22 3NE UK; Nottingham Clinical Trials Unit, C floor South Block, QMC, Nottingham, NG7 2UH UK; School of Nursing, Queens Medical Centre, University of Nottingham, B Floor, South Block, Nottingham, NG7 2UH UK; Faculty of Medicine & Health Sciences, University of East Anglia, Queens Building, Norwich Research Park, Norwich, NR4 7TJ UK; Acquired Brain Injury Service, Acorn Suite, 1829 Building, Countess of Chester Health Park, Liverpool Road, Chester, CH2 1HJ UK; Service User Representative, Kragujevac, UK

**Keywords:** Traumatic brain injury, Memory, Cognitive rehabilitation, Randomised controlled trial, Cost-effectiveness

## Abstract

**Background:**

Impairments of memory are commonly reported by people with traumatic brain injuries (TBI). Such deficits are persistent, debilitating, and can severely impact quality of life. Currently, many do not routinely receive follow-up appointments for residual memory problems following discharge.

**Methods/Design:**

This is a multi-centre, randomised controlled trial investigating the clinical and cost-effectiveness of a group-based memory rehabilitation programme. Three hundred and twelve people with a traumatic brain injury will be randomised from four centres. Participants will be eligible if they had a traumatic brain injury more than 3 months prior to recruitment, have memory problems, are 18 to 69 years of age, are able to travel to one of our centres and attend group sessions, and are able to give informed consent. Participants will be randomised in clusters of 4 to 6 to the group rehabilitation intervention or to usual care. Intervention groups will receive 10 weekly sessions of a manualised memory rehabilitation programme, which has been developed in previous pilot studies. The intervention will include restitution strategies to retrain impaired memory functions and compensation strategies to enable participants to cope with their memory problems. All participants will receive a follow-up postal questionnaire and an assessment by a research assistant at 6 and 12 months post-randomisation. The primary outcome is the Everyday Memory Questionnaire at 6 months. Secondary outcomes include the Rivermead Behavioural Memory Test-3, General Health Questionnaire-30, health related quality of life, cost-effectiveness analysis determined by the EQ-5D and a service use questionnaire, individual goal attainment, European Brain Injury Questionnaire (patient and relative versions), and the Everyday Memory Questionnaire-relative version. The primary analysis will be based on intention to treat. A mixed-model regression analysis of the Everyday Memory Questionnaire at 6 months will be used to estimate the effect of the group memory rehabilitation programme.

**Discussion:**

The study will hopefully provide robust evidence regarding the clinical and cost-effectiveness of a group-based memory rehabilitation intervention for civilians and military personnel following TBI. We discuss our decision-making regarding choice of outcome measures and control group, and the unique challenges to recruiting people with memory problems to trials.

**Trial registration:**

ISRCTN65792154; Date: 18 October 2012

## Background

Impairments of memory are the most common cognitive deficits reported by people with traumatic brain injuries (TBIs), affecting 40 to 60% of patients [1, 2]. These memory problems are not only persistent, but are debilitating and difficult to treat [3]. Memory deficits may also affect the extent to which patients engage with other interventions and rehabilitation. The safety of such patients can also be compromised, making them vulnerable citizens in the home (for example, forgetting to turn the stove off), community (for example, forgetting road rules), and work (for example, forgetting important documents) settings. Memory problems consequently have a devastating effect on the psychological wellbeing of the individuals and others around them [4].

Costs of morbidity due to TBI are incurred by the healthcare system and those outside it (in terms of loss of productivity due to short-term sick leave and early retirement), and through non-medical costs (for example, transformations of house or work environments, etc.). In addition, informal care by family or friends can dominate the costs of care for affected individuals. For TBI, the direct medical costs and indirect costs were estimated at $60 billion in the United States in 2000 [5]. The full costs of dealing with memory problems caused by TBI in the UK are not known. Care costs escalate when an intervention is provided on an inpatient basis, but Salazar *et al*. [6] demonstrated that the benefits of inpatient and home cognitive rehabilitation programmes for TBI, in terms of return to duty (for military personnel) or employment, were similar.

Cognitive rehabilitation is a structured set of therapeutic activities designed to retrain an individual’s memory and other cognitive functions. A narrative review [7] found cognitive rehabilitation to be beneficial for treating cognitive deficits following brain damage. There are recommendations for the provision of cognitive rehabilitation for people with acquired brain injuries (for example, European Federation of Neurological Societies Guidelines on cognitive rehabilitation [8]; National Service Framework for Long term Conditions [9]). However, recommendations are always qualified by statements that highlight the need for more research, to support the recommendations.

Some randomised controlled trials (RCTs) have demonstrated the effectiveness of cognitive rehabilitation following brain injuries. These have mainly focussed on attention, executive functions, and visual neglect, but memory rehabilitation has not been sufficiently researched [10]. Most evidence for memory rehabilitation comes from single-case experimental design studies and controlled clinical trials. The few RCTs and quasi-RCTs in this area have offered some support for the effectiveness of intervention. Wilson *et al*. [11] examined an external memory aid, Neuropage. This enabled participants to achieve more memory-related goals than when it was not available. Doornhein and de Haan [12] reported that patients who received a memory training programme performed significantly better than those in a pseudo-treatment control group on trained memory tasks, but no differences were observed on subjective ratings of everyday memory functions. Kaschel *et al*. [13] reported that imagery mnemonics significantly improved delayed recall of verbal material and reduced observer-rated reports of memory failures. However, systematic reviews on memory rehabilitation have not found evidence to support or refute the effectiveness of such programmes [14, 15]. This lack of evidence is partly due to the paucity of well-designed trials and has led a recent meta-analysis to conclude that ‘the results for memory rehabilitation are mixed and weak’ [10] (p.33). These authors suggested that ‘researchers need to reduce reliance on single-subject and single group designs’ (p.34) and recommended more RCT evidence, a view supported by others [16]. At a symposium on disorders of memory, Wilson called for ‘better evaluation of memory rehabilitation programmes’ [17] (p.e4-5). This is a conclusion that our systematic reviews of memory rehabilitation following TBI [10], stroke [18], multiple sclerosis [19] have also reached. A small scale RCT (n = 72) was conducted to evaluate a group memory rehabilitation programme [20]. Patients with memory problems were randomly allocated to one of three group treatment programmes: compensation strategy training, restitution, or a self-help attention placebo control. The results showed that there were no statistically significant differences in outcome. However, the trend in the results and the qualitatively analysed participant feedback interviews [21] indicated the interventions seemed worthy of further evaluation. These studies provided feasibility and pilot data for the present study.

Currently, TBI patients with memory problems do not routinely receive follow-up rehabilitation after the early intensive phase, even though their abilities and needs may change once discharged from clinical services. This is mainly due to the current lack of evidence of clinical and cost-effectiveness of the intervention, and resource limitations. This study seeks to address these concerns.

## Methods/Design

### Trial objectives

The primary objective is to determine whether attending a group memory rehabilitation programme is associated with subjective reports of improved management of memory in daily life when compared to a usual care (UC) control. The secondary objectives are to assess whether the intervention is associated with improvements in ‘objectively’ assessed memory abilities, participants’ ability to achieve individually set goals, health-related quality of life, and cognitive, emotional and social wellbeing. Lastly, the cost-effectiveness of the intervention will also be investigated.

### Trial design

This is a multi-centre, parallel group, randomised controlled trial (RCT).

### Site and participant recruitment

The study will be conducted in at least four centres in the UK. New centres will be activated as old ones shut down due to their participant pools being exhausted.

Participants are identified through NHS hospitals, rehabilitation centres and charities (for example, military or head injury charities). A letter will be sent to individuals who are identified as potential participants by a member of the clinical team; this letter will include a patient information sheet, a consent form and a paid reply envelope. If the potential participant is interested in taking part, he or she is requested to complete the slip and return it in the envelope directly to the Assistant Psychologist (AP). Self-referral will also be possible via public-facing information on the study website, newsletters and posters. Participants recruited via this route will be made aware that their GP may be contacted to confirm the TBI diagnosis. Recruitment is planned to cover a 25-month period.

### Informed consent

Written informed consent will be obtained by the AP. We will explain to participants that their participation is entirely voluntary and they are free to withdraw at any time; in the event of their withdrawal, any data collected up until that point would be kept by the research team. Participants will also be asked to allow the research team to have access to their clinical notes to obtain information on their clinical diagnosis, severity of injury (Glasgow Coma Scale score), time since injury, and other medical conditions. They will be asked whether they consent to a follow-up interview to assess treatment acceptability and will be informed that if allocated to the intervention group, sessions may be video-taped to ensure treatment fidelity. The GPs of consenting participants will be sent a letter to inform them of their patients’ involvement in the trial.

### Inclusion criteria

Patients are eligible for the trial if they i) were admitted to hospital with a TBI more than 3 months prior to recruitment, ii) report having memory problems as assessed at baseline, iii) are 18 to 69 years of age, iv) are able to travel to one of the study centres and attend group sessions, and v) give informed consent.

### Exclusion criteria

Potential participants will be excluded if they i) are unable or unsuitable to engage in group treatment if allocated, ii) are involved in other psychological intervention studies, or iii) have impairment of language, as assessed at baseline.

### Initial screening assessment

At the first appointment, the AP will explain the study and make clear that the initial screening assessments are required to check that the patient meets the inclusion criteria. The AP will obtain informed consent and conduct the initial assessments. The following assessments will be conducted at screening:

1. The Everyday Memory Questionnaire-patient version (EMQ-p [22]; a subjective measure of the frequency of memory failures in daily life. We will be using a modified version of the EMQ, which has two sections: (i) frequency of forgetting of individual items and (ii) importance of each individual item to the patient.
2. The Rivermead Behavioural Memory Test-3rd edition (RBMT-3; [23]) an ecologically valid measure of memory ability. The RBMT-3 has been used in previous studies and is the version currently used clinically.
3. The Sheffield Screening Test for Acquired Language Disorders (SST; [24]) used to assess language ability. We will use a cut-off score <17 to determine eligibility.
4. Premorbid level of intellectual functioning will be estimated using the National Adult Reading Test (NART; [25]).
5. General Health Questionnaire (GHQ-30; [26]) a measure of mood.

Potential participants will be eligible to take part if they meet the following requirements:

1. score 24 or more on Section 1 (frequency) of the EMQ OR.
2. score below the 25th percentile on the RBMT-3.

Those who do not meet the inclusion criteria will be notified by letter to thank them for their interest in the study and a brief report of their test results will be provided if requested. Those who meet the inclusion criteria will be phoned to arrange a second assessment session if they are willing to continue. We will also check whether they can nominate a relative or friend who knows them well who would be willing to complete two questionnaires about the participant’s memory.

### Second assessment

The purpose of this assessment, conducted 2 weeks (+/- 1 week) after the first assessment, is to achieve the following:

1. set the short- and long-term goals that individual participants would like to achieve by the end of the study (all participants set one short term and long term goal up to a maximum of five).
2. complete the EQ5D (a health-related quality of life measure [26]).
3. complete the service-use questionnaire.
4. check their availability in the event they are assigned to the intervention group.
5. collect the EMQ-r, completed by the carer/relative.

### Participant outcome assessments

Outcome assessments will occur at 6 and 12 months after randomisation. The NHS number will be supplied to the Medical Research Information Service to allow a mortality check prior to follow-up.

The primary outcome measure will be the EMQ-patient version [22]. The study will use the frequency component as the primary outcome measure and the importance component will be used to develop a scoring mechanism to develop an assessment, which takes frequency and importance into account.

Participants will receive a questionnaire pack by post, and will be requested to return this to the Nottingham Clinical Trials Unit (NCTU) as soon as possible; however, participants do have the option of requesting help in completing these questionnaires if necessary. If questionnaires have missing items or are not returned, participants will be telephoned to obtain the missing information. The questionnaire pack will consist of: the modified EMQ (patient and relative version), GHQ-30, European Brain Injury Questionnaire [27] (patient and relative versions), EQ5D [28], and a bespoke service-use questionnaire. A research assistant, who is unaware of the group allocation, will conduct the RBMT-3 and assess goal attainment.

### Minimisation of bias

The participants and APs will not be blind to the allocated treatment. The primary outcome is participant completed and sent back directly to the data entry team. The RBMT and assessment of goal attainment (secondary outcomes) will be conducted by the RA in person. To prevent unblinding, the RA will request participants not to discuss any aspect of being involved with the study. The RA will also be required to guess the treatment allocation for each participant and this will be compared later to the actual allocation, to determine the degree of unblinding.

### Randomisation

Participants will be randomised in clusters of four to six. Once four to six participants have been identified and consented, they will be randomly allocated, as a group, to intervention or usual care (UC) (1:1 ratio). The randomisation will be based on a computer generated pseudo-random code using random permuted blocks of randomly varying size, created by the NCTU in accordance with their standard operating procedure and held on a secure server. The randomisation will be stratified by study site. Access to the sequence will be confined to the NCTU IT Manager. Investigators will access the allocation for each group by means of a remote, internet-based randomisation system developed and maintained by the NCTU. The sequence of treatment allocations will be concealed from the study statistician until all interventions have all been assigned and recruitment, data collection, and all other study-related assessments are complete.

### Duration of participant participation

**Figure 1:**
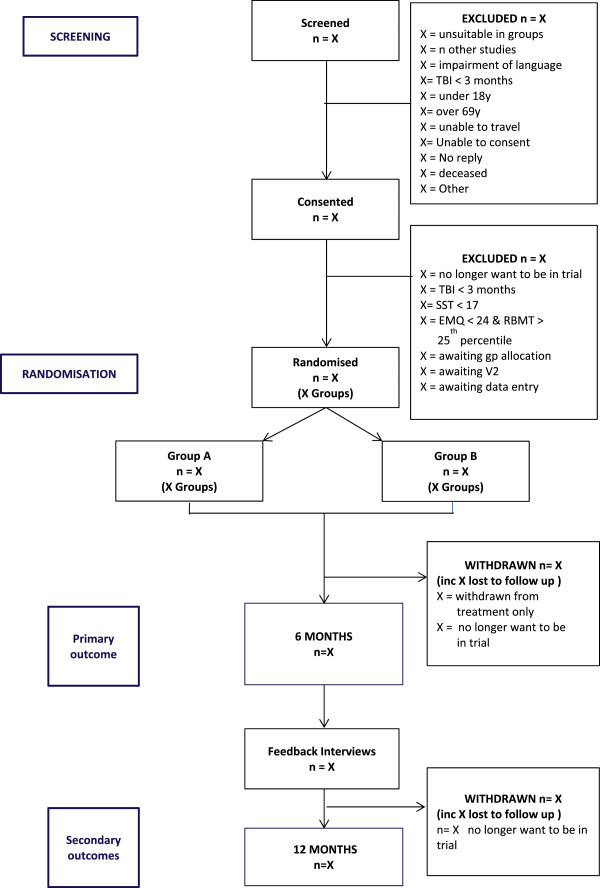
**CONSORT diagram.**

Figure 1 shows the expected progress of the study. Participants are in the study for approximately 13 months from the initial screening assessment (12 months from randomisation). Participants will leave the study when they have completed the 12-month follow-up.

### Group-based memory intervention

Each group led by an AP will consist of 4 to 6 participants. Participants will receive 10 group memory-rehabilitation sessions (1.5 hours long, once a week for 10 weeks), following a treatment manual, which was developed and tested in the previous study (a description of the manual has been published [14, 20]). The original manual has been revised following extensive consultation and feedback from participants. Qualitative research [22] also found that the group format, composition, and duration were acceptable to participants, and delivery of the intervention was feasible. The intervention will include restitution strategies to retrain memory functions, including attention retraining and strategies to improve encoding and retrieval. Compensation strategies will be taught, including internal mnemonics (such as chunking, use of first letter cues, rhymes), use of external devices (such as diaries, mobile phones, calendars), and ways of coping with memory problems. The importance of ‘errorless learning’ [29] will also be taught.

### Control group (usual care)

Participants will receive their usual clinical care. The majority of participants will no longer be receiving any formal rehabilitation. They may be attending self-help groups or services from charities supporting people with head injuries, for example, Headway.

All other clinical services will be provided as usual for both groups. This may include referral to employment rehabilitation services, self-help groups or support from specialist charities, such as Headway. Any additional input (including psychological or medical interventions) participants receive during the study will be noted from the service use questionnaire.

### Compliance with interventions

The assistant psychologist records whether participants attend each of the treatment sessions and the reasons if sessions are not attended. To ensure the fidelity of the intervention, the content of treatment will be described and analysed. This will be achieved by video recording 20 intervention sessions. Sessions will be purposively pre-selected for recording in order to include sessions from the start, middle and end of the ten-week course and recordings will be made across the intervention period. Practices for video-recording will draw upon guidance on minimizing intrusiveness of the recording [30, 31]. Methods used in previous work will be drawn on to analyse the content of training within rehabilitation contexts [32, 33]. Two independent assessors will separately analyse the video recordings using a customized score sheet to capture a variety of key elements spanning all aspects of the intervention. Assessors will code these factors as present or absent over a series of time intervals. This method has previously been used in the pilot study and was able to determine treatment fidelity without disrupting the group sessions [34].

### Sample size and justification

The sample size calculation is based on the primary outcome measure (EMQ-p frequency total score) at six months post-randomisation. The main study aim is to detect a minimum clinically relevant difference in mean EMQ-p frequency total score of 12 between the memory intervention group and the usual care group. A 12-point difference on this measure was deemed to be a clinically significant change based on our pilot data [20] and clinical interviews. A common standard deviation of 21.9 from the pilot gives an effect size of 0.55. A type 1 error of 0.05 and power of 90% were used for the calculation. A fixed effects model at the level of the four centres is assumed, with 10% of the total variation due to between-centre variation. The participants are cluster randomised into groups of 6 at the second level and a random effects model will be used with a small intracluster correlation coefficient assumed (ICC = 0.1). This ICC is likely to be small because within each centre the therapist, intervention, and delivery location do not vary. Using the ‘Optimal Design’ software with these parameters, the calculation gives 10 groups per centre. Data from the pilot study and taking account that the control group only receives usual care suggests a possible dropout rate of 20%, so 26 groups of each intervention will be required or 312 participants in total. Based on our pilot study, we estimate we will need to screen 400 participants to recruit the required 312. Clinicians at the four centres have indicated that this is an achievable target in the timeframe proposed.

### Statistical analysis

Database lock will take place once all data entries have been checked and sufficient verification processes have been completed. Analyses will be completed with Stata version 11.2 or above. The psychometric properties of the instruments used will be evaluated when sufficient baseline data have been collected. The baseline characteristics will be described using appropriate summary statistics to examine balance between the two randomised groups.

The primary analysis will use a multi-level linear model with baseline EMQ score as a covariate, centre as a fixed effect and random effects, which take appropriate account of clustering according to allocated group [35, 36]. The between group comparison will be presented as the difference in mean EMQ-p score at 6 months between the two groups with a 95% confidence interval [35, 36]. Participants will be analysed as randomised (Intention to Treat) regardless of adherence with allocation. It is planned that the primary analysis will be based on participants with available data with no imputation for participants with missing outcomes. The distributions of raw outcome scores and residuals will be examined and the data suitably transformed or a non-parametric analysis employed if necessary.

Supportive analyses for the primary outcome will include imputation of missing data exploring different scenarios for the missing data, accounting for non-compliance with the group memory rehabilitation programme and adjusting for any characteristics assessed at baseline with an observed imbalance between the two groups.

All secondary variables will be presented using appropriate descriptive statistics and analysed with appropriate regression models using the same techniques as for the primary outcome. Differences between the two groups will be presented with 95% confidence intervals. All secondary analyses will be interpreted with caution as the sample size calculation is based on the primary outcome. The analyses for all the outcomes will be repeated with the 12 month follow-up data following similar distribution checks.

Full details of all statistical analyses will be given in a statistical analysis plan, which will be finalised prior to database lock.

### Health economic evaluation

The cost-effectiveness will be assessed from the perspective of the UK NHS and personal social services. The costs associated with the intervention will be determined by calculating the cost of staff time, materials, etc. used in providing the intervention. These will be compared with changes in the number of visits to GPs, hospital, prescribed medication, and social services contacts in the intervention and control groups during the investigation. The costs will be compared with the outcomes generated and a series of incremental cost-effectiveness ratios computed, including a cost/Quality Adjusted Life Year (QALY) analysis - based on changes in EQ-5D. A series of one-way sensitivity analyses will be undertaken to determine the extent to which baseline findings will change in light of parameter variation. Given the limited time duration of the study and follow-up, a decision analytic model will be constructed to determine the cost-effectiveness of the intervention from a lifetime perspective, a series of scenarios will be constructed to reflect the extent to which differential outcomes can be predicted to continue over longer time periods, using expert opinion and information available in the literature. A probabilistic sensitivity analysis will be carried out to determine the extent to which the intervention can be regarded as representing value for money.

### Assessment of safety and adverse events

The adverse event risks of taking part in the study have been assessed. A part of the baseline assessment is to assess the memory of the participants such that they may become aware of memory problems that they did not know that they had. As a result, the main risk associated with this intervention is distress caused by the realisation that their memory is not as good as they had thought. However, first, distress caused in this way is considered very unlikely; and, second, any distress caused is likely to be mild. In addition, for the intervention group, this is also dealt with during the course of the intervention and the group therapy will address this on a participant by participant basis. So overall the risk has been assessed as negligible.

### Participants who withdraw

No withdrawal criteria have been specified, and participants have the right to withdraw from the study at any time. The reasons for leaving the study will be recorded, but participants are not obliged to give reasons. Participants will be assured that withdrawal will not affect the care they receive. They will be informed at the start of the study that data collected up to the point of withdrawal will be retained and may be used in the final analysis. There will be no replacement of participants who withdraw.

All reasonable attempts will be made to contact any participant lost to follow-up during the course of the study in order to complete assessments.

### Feedback interviews

A feedback interview will be conducted within 2 months of the 6 month appointments, with 32 purposefully selected and willing participants: 16 from each group. This will be 4 intervention and 4 control participants, from each participating centre. The selection strategy will be designed to include participants with varying levels of memory impairments, and with varying social situations. The interviews will be conducted by a second RA (RA2) who was not involved with the participant’s assessment or treatment, thereby reducing social desirability response bias. The RA2 will become aware of the group allocations during the interview so will not be blind to the intervention. The interview will be audio recorded using a digital recorder, transcribed, and analysed using a thematic analysis (following the protocol prescribed by Braun and Clarke [37]). Participant consent for the interviews will be sought separately. The interviews will provide important feedback on participants’ perception of progress over time and for those in the intervention groups, the quality of the interventions provided, and as such will serve as a process measure. Insights from this qualitative data and analysis will serve to inform developments of the intervention programme in the future and to generate user-oriented proposals about areas for further investigations. For those in the control group the interviews will provide confirmation of the nature of usual care received.

### Criteria for terminating the study

The study maybe stopped as a whole because of a change in opinion of the Research Ethics Committee (REC) or safety concerns or issues with study conduct at the discretion of the sponsor.

### Trial management

A Trial Management Group (TMG) will be convened and meet regularly. This group will be in charge of the everyday running of the trial. The Trial Steering Committee (TSC) will oversee the conduct of the study and will have an independent Chair. A service user representative and a member from one of the military charities will also be invited to join this group. It will advise on recruitment strategies, monitor progress with recruitment, and check adherence to the study protocol. Observers from the National Institute for Health Research - Health Technology Assessment (NIHR HTA) programme (the funder) will be invited to TSC meetings. The Data Monitoring Committee (DMC) will be an independent group, the members of which have no other involvement with the study. Members of this committee will include rehabilitation professionals and an experienced study statistician. It will safeguard the interests of trial participants, with particular reference to safety and the efficacy of the intervention, monitor the overall progress and conduct of the trial and assist and advise the Investigators so as to protect the validity and credibility of the trial.

### Service user involvement

Our service user representative has had experience of rehabilitation in NHS services and has taken part in the previous study. Their role will be to advise on recruitment and dissemination options, and will contribute to the development of the intervention manual and the lay summary of the project. This service user will sit on the TSC. Other service user representatives will be recruited to the TSC and DMC from relevant charities (for example, Headway and The Soldiers’ Charity). Service user involvement will contribute to: team meetings and project management decisions, project approval through REC, recruitment and consent (contribute to the development of participant information sheets), data gathering (through developing patient information leaflets explaining the survey tools where appropriate), interpretation of findings (through the development of recommendations for practice and patient information leaflets about therapy), and dissemination of the findings through existing networks.

### Definition of a protocol deviation

A protocol deviation is an unanticipated or unintentional divergence or departure from the expected conduct of a study inconsistent with the protocol, consent document or other study procedures. All protocol deviations will be recorded on the electronic case report form (eCRF) by local investigator staff.

### Ethical approval

Ethical approval was obtained from the NRES Committee East Midlands - Nottingham 1 on 21.09.12. (ref: 12/EM/0324).

## Discussion

This study was conceptualised in response to a commissioned call for ‘proposals concerning people needing physical or psychological rehabilitation following trauma in a military or civilian context’ from the Health Technology Assessment (HTA) Programme [38]. Based on our pilot work [14] and feedback from participants [21], we decided to combine restitution and compensation approaches in the intervention. We also decided to have a usual care control group, as having a self-help control group was difficult to organise and facilitate.

Our choice of primary outcome measure was a point of contention, with some of our reviewers suggesting ‘objective’ measures of memory, others recommending the use of goal attainment scaling, and yet others agreeing that the primary outcome had to be a subjective report of memory function in daily life. We have retained both an objective measure of memory and a measure of goal attainment, but these are secondary outcomes. Most objective measures have poor ecological validity. Goal attainment poses challenges in setting goals that can be precisely measured and the changeable nature of some of the goals, given that our outcomes are measured 6 and 12 months after the goals were set.

We anticipate that one of the biggest challenges to recruit to this study will be potential participants’ memory problems themselves. Even those interested in taking part in this trial when they receive our invitation letter may forget to respond. To address this, we have sought an amendment to our original ethics approval to include a single phone call to follow-up non-responders to the invitation letter to enquire whether they remember receiving the letter and whether they would like to participate.

## Trial status

The first centre was open to recruitment on the 6 February 2013 and the first participant consented on 20 February 2013. At the time of preparing this manuscript, 166 people have consented, and 85 have been randomised. Recruitment is due to finish in March 2015.

## Abbreviations

AP: assistant psychologist
CI: chief investigator
CLRN: Clinical Local Research Network
DMC: data management committee
eCRF: electronic case record form
EMQ: Everyday Memory Questionnaire
GHQ: General Health Questionnaire
HTA: Health Technology Assessment
IRAS: Integrated Research Application System
NART: National Adult Reading Test
NCTU: Nottingham Clinical Trials Unit
NHS: National Health Service
NIHR: National Institute for Health Research
QALY: quality adjusted life years
RA: research assistant
RBMT: Rivermead Behavioural Memory Test
REC: research ethics committee
SST: Sheffield Screening Test
TBI: Traumatic Brain Injury
TMG: trial management group
TSC: trial steering committee.
